# Editors’ Note

**DOI:** 10.63144/ijt.2025.6709

**Published:** 2025-06-12

**Authors:** Ellen R. Cohn, Jana Cason

## Journal Overview

The International Journal of Telerehabilitation (IJT) is a biannual journal dedicated to advancing telerehabilitation by disseminating peer-reviewed information about current research and practices. IJT is indexed by PubMed and Scopus. IJT is published via Open Journal Systems (OJS) and is now sponsored by Hawaiʻi Pacific University. This institutional support enables IJT to be subscription-free and require no author fees.

IJT enjoys a diverse international audience due to the expanding global relevance of telemedicine, telehealth, and telerehabilitation. Recently, we are pleased to publish work by authors from Australia, Brazil, Canada, Colombia, Egypt, Germany, India, Indonesia, Malaysia, the Netherlands, Poland, Saudi Arabia, Taiwan, Thailand, Ukraine, and the United States. We hope you enjoy reading this new issue of IJT!

We are grateful to our diligent and generous peer reviewers who donate their time and hail from multiple disciplines. Their suggestions invariably elevate the work of our most experienced authors. Our reviewers are not named herein, to preserve the anonymity of their reviews.

## New Beginnings

Beginning with the current issue, IJT has been “rehomed” to Hawaiʻi Pacific University, where Dr. Jana Cason is a full-time faculty member in the Graduate College of Health Sciences. We are grateful for the warm welcome from IJT’s new publisher, Sabrina Thomas, EdD, Director of Library and Learning Commons, Hawaiʻi Pacific University and the exceptional technical support provided by Daniel S. Brown, MSI, PhD, Collection Development and Scholarly Communications Librarian, Hawaiʻi Pacific University. We appreciate the support of others at Hawaiʻi Pacific University including Jennifer Walsh, Senior Vice President of Strategic Initiatives and Chief Strategy Officer; Brenda Jensen, Acting Provost and Professor of Biology; Tricia Catalino, Dean, Graduate College of Health Sciences and Professor of Physical Therapy; and Tracey Recigno, Doctor of Occupational Therapy Program Director and Assistant Professor of Occupational Therapy.

## Mahalo to IJT’s First Home

Special appreciation is due to recognize IJT’s seamless transition from the University of Pittsburgh to Hawaiʻi Pacific University. Richard L. Hoover, Interim Director of the Office of Scholarly Communication and Publishing at University of Pittsburgh Library System (ULS) and his staff, (most recently Library Publishing Specialist Marin Dremock), have generously and expertly supported IJT during its transition. We are grateful to these outstanding professionals. Their work will continue to be foundational for IJT and is preserved in the University of Pittsburgh Library System (ULS) Archive. As well, we acknowledge Joseph K. Ruffing at the University of Pittsburgh who designed the first IJT journal website and article template in 2008 that continues to visually define the Journal.

## Next Issue: telerehab.hpu.edu

All former issues and the current issue are now located at: telerehab.hpu.edu. The next issue will be published in October 2025. IJT will open for new submissions on or about July 1, 2025 through August 1, 2025.

Mahalo nui loa,

Jana Cason, DHSc, OTR/L, FNAP, FAOTA

Ellen R. Cohn, PhD, CCC-SLP, ASHA-F

IJT Co-Editors-in-Chief

